# COUP-TFII is required for morphogenesis of the neural crest-derived tympanic ring

**DOI:** 10.1038/s41598-017-12665-0

**Published:** 2017-09-28

**Authors:** Wen-Hsin Hsu, Chun-Ming Chen, Li-Ru You

**Affiliations:** 1 Institute of Biochemistry and Molecular Biology, National Yang-Ming University, Taipei, 112 Taiwan; 2 Department of Life Sciences and Institute of Genome Sciences, National Yang-Ming University, Taipei, 112 Taiwan; 3 VYM Genome Research Center, National Yang-Ming University, Taipei, 112 Taiwan

## Abstract

Chicken Ovalbumin Upstream Promoter-Transcription Factor II (COUP-TFII) plays pivotal roles in cell growth, cell differentiation, and cell fate determination. Although genome-wide studies have identified COUP-TFII binding on gene sets mainly involved in neural crest cell (NCC) development and craniofacial morphogenesis, the direct functional connection between COUP-TFII and NCCs *in vivo* has not been well characterized. In this study, we show that COUP-TFII is expressed in the subpopulation of NCCs and its derivatives, and targeted ablation of *COUP-TFII* in mouse NCCs results in markedly shortened and bifurcated tympanic rings, which in turn disturb the caudal direction of external acoustic meatus invagination. However, formation of the manubrium of the malleus (MM) in *Wnt1-Cre*/+*;COUP-TFII* ^*flox/flox*^ mice is not perturbed, suggesting that the rostral half of the tympanic ring is sufficient to support proper MM development. Interestingly, we found that loss of *COUP-TFII* up-regulates Sox9 in the tympanic ring primordium and affects the distribution of preosteoblasts before mesenchymal condensation. Together, our results demonstrate that COUP-TFII plays an essential role in regulating the patterning of the NCC-derived tympanic ring.

## Introduction

Neural crest cells (NCCs) are a multipotent, migratory cell population that is unique to vertebrates and is generated transiently during embryonic development. NCCs originate from neural folds at the dorsal part of the closing neural tube, undergo an epithelial-to-mesenchymal transition, delaminate from the neuroepithelium, and migrate extensively to generate numerous derivatives. According to their emigration position along the anteroposterior axis of the embryo, NCCs can be divided into four main functional domains—cranial, cardiac, vagal and sacral, and trunk—each of which possesses distinct differentiation capacities. Of these, cranial NCCs are capable of differentiating into bone and cartilage tissues and constitute the majority of craniofacial structure^1^. In addition, the bones of skull develop through endochondral or intramembranous ossification, both of which begin with mesenchymal condensation. During endochondral ossification, condensed mesenchymal cells first differentiate into chondrocytes to form a cartilage intermediate that is subsequently replaced by bone. By contrast, during intramembranous ossification, mesenchymal cells directly differentiate into osteoblasts^2^.

The Sry-related transcription factor Sox9 and runt-related transcription factor 2 (Runx2/Cbfa1) are crucially involved in skeletal development. Mutations in human SOX9 and RUNX2 genes cause the heritable skeletal disorders campomelic and cleidocranial dysplasia, respectively^3–6^. Targeted ablation of *Sox9* in mice shows that Sox9 not only is required for promoting the commitment of mesenchymal cells to the chondrogenic cell lineage but also serves as a central player in maintaining the lineage fate and successive differentiation program of growth plate chondrocytes^7–9^. Runx2 is imperative for osteoblast cell fate determination and regulates the expression of many osteoblastic genes. Therefore, Runx2 is indispensable for osteoblast differentiation during both endochondral and intramembranous ossification^10–13^. Based on the expression profiles of Sox9 and Runx2 at the mesenchymal condensation stage, a binary molecular code is proposed to predict the identity of skeletal tissues^14^. Before overt skeletogenesis, both Sox9 and Runx2 are expressed in dual-potential osteochondral progenitors. During intramembranous bone formation, the expression of Sox9 is down-regulated and the expression of Runx2 is up-regulated in mesenchymal condensation. On the contrary, Sox9 expression is up-regulated and Runx2 expression is down-regulated during chondrogenic mesenchymal condensation^14,15^. Nevertheless, Runx2 expression is subsequently up-regulated, which facilitates later endochondral bone formation through activating chondrocyte maturation and osteoblast specification and differentiation in the perichondrium^12,16^.

Chicken Ovalbumin Upstream Promoter-Transcription Factor II (*COUP-TFII*, also known as *Nr2f2*) is an orphan member of the steroid/thyroid hormone receptor superfamily. Previous studies showed that COUP-TFII can function as a transcriptional repressor through direct binding to COUP-TFII response elements or a transcriptional activator by associating with specific transcriptional factors to regulate their target genes in diverse cellular contexts^17,18^. COUP-TFII is expressed abundantly during the early embryonic stage. Loss of *COUP-TFII* results in lethality at embryonic day (E)10 with severe defects in angiogenesis and heart development, demonstrating the crucial role of COUP-TFII during mouse embryogenesis^19^. Conditional ablation of *COUP-TFII* further revealed that COUP-TFII makes different contributions to the development of various organs, including cell fate specification in venous and lymphatic vasculature^20,21^, atria of the heart^22^, and metanephric mesenchyme^23^; endocardial and epicardial epithelial-mesenchymal transformation^24^; differentiation of Leydig cells^25^; and cell proliferation and apoptosis in granule cell precursors of the cerebellum^26^. COUP-TFII can also fine-tune the lineage-specific differentiation of mesenchymal stem cells to osteogenic and myogenic lineages at the expense of adipocyte and chondrocyte development, both *in vivo* and *in vitro* ^27^.

Recently, genome-wide epigenomic profiling of chromatin landscapes, gene expression, and cis-element sequence analysis of human embryonic stem cell-derived NCCs revealed that TFAP2A, COUP-TFI, and COUP-TFII simultaneously co-occupy human NCC enhancers and facilitate establishment of a transcriptionally active chromatin enhancer state. The functional annotation of TFAP2A and COUP-TFI/COUP-TFII co-bound regions uncovered a strong association with NC gene expression, cranial NCC-derived structures, and craniofacial anomalies, suggesting that COUP-TFII regulates NC development and craniofacial morphogenesis^28,29^. However, the *in vivo* physiological function of COUP-TFII in NC development has not been well studied. Here, we show that COUP-TFII is expressed in NCCs and their derivatives. Targeted ablation of *COUP-TFII* in premigratory NCCs by *Wnt1-Cre* transgenic mice^30^ dramatically shortens the tympanic ring with a bifurcated end. Loss of *COUP-TFII* up-regulates the expression of Sox9 in the tympanic ring primordium before mesenchymal condensation and affects the distribution of preosteoblasts, uncovering an essential role of COUP-TFII in the patterning of tympanic ring development. Therefore, our study provides new insights into the role of COUP-TFII in NCC-derived intramembranous bone formation.

## Results

### COUP-TFII is expressed in the NCC-derived subpopulation

**Figure 1:**
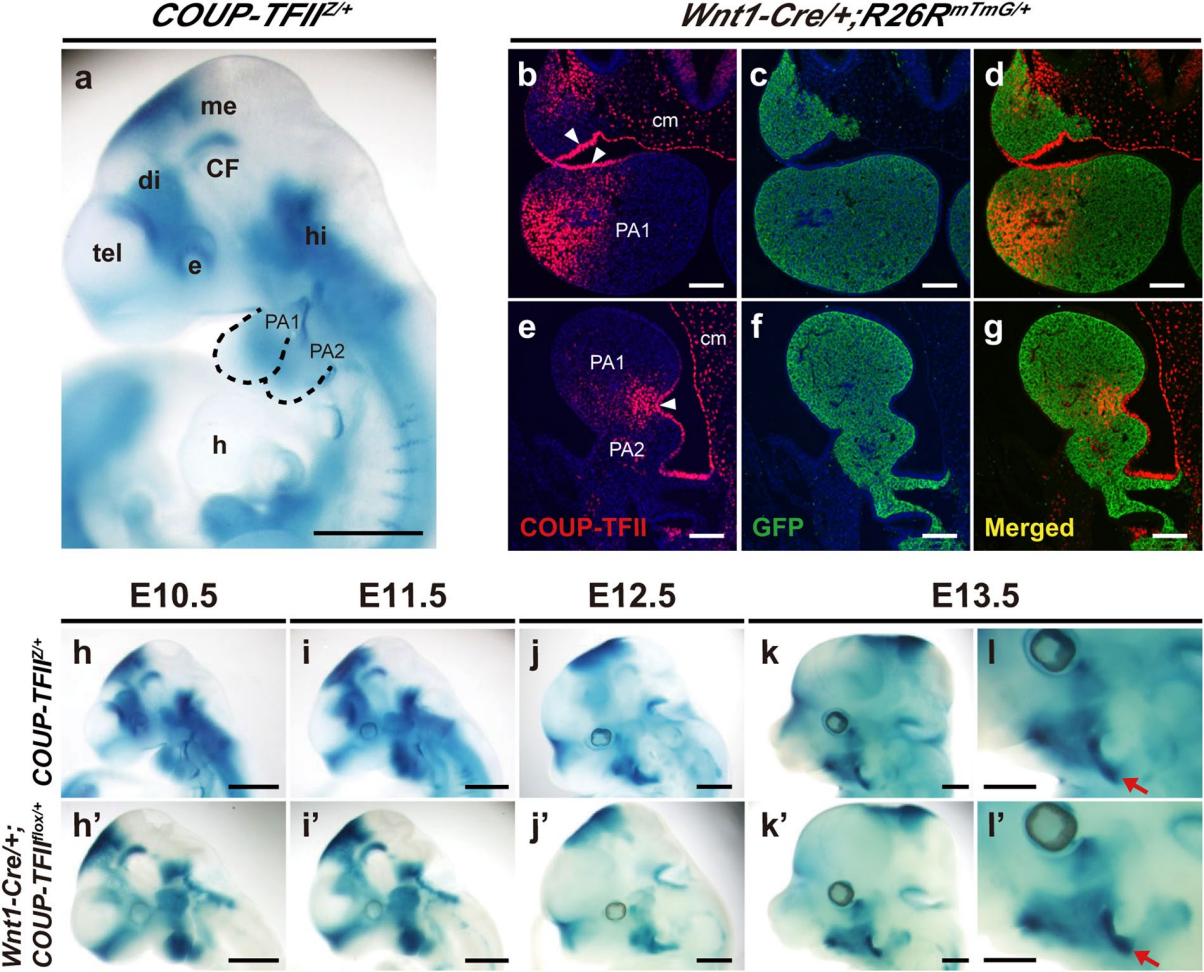
COUP-TFII is expressed in the NCC-derived subpopulation. (**a**) Lateral view of a whole-mount X-Gal-stained E10.5 *COUP-TFII* ^*Z*/+^ embryo. COUP-TFII was highly expressed in the caudal telencephalon, posterior two-thirds of the diencephalon, diencephalon-mesencephalon boundary, cephalic flexure, hindbrain, and craniofacial ganglia. A moderate level of COUP-TFII was specifically detected in the posterior part of PA1 and anterior part of PA2. COUP-TFII expression was detected in the upper frontonasal region, albeit at a very low level. Dashed lines demarcate the boundaries of PA1 and PA2. n = 13. (**b**–**g**) Immunofluorescence analyses of COUP-TFII and GFP expression in transverse (**b**–**d**) and sagittal (**e**–**g**) sections of E10.5 *Wnt1-Cre*/+*;R26R* ^*mTmG/*+^ embryos. COUP-TFII was detected in cephalic mesenchyme (cm), pharyngeal endoderm (arrowheads), and the subpopulation of mesenchymal cells in PA1 and PA2 (**b** and **e**, red). GFP-positive (i.e., NCC-derived) cells (**c** and **f**, green) appeared in most mesenchyme of PA1 and PA2. COUP-TFII expression was observed in GFP-positive mesenchymal cells of PAs. Nuclei were stained with 4,6-diamidino-2-phenylindole (DAPI, blue). n = 3. (**h**–**l”**) Spatiotemporal expression profile of COUP-TFII in the NCC lineage. Lateral views of whole-mount X-Gal-stained *COUP-TFII* ^*Z*/+^ (E10.5, n = 13; E11.5, n = 4; E12.5, n = 7; E13.5, n = 2) (**h**–**l**) and *Wnt1-Cre*/+*;COUP-TFII* ^*flox*/+^ (E10.5, n = 3; E11.5, n = 6; E12.5, n = 5; E13.5, n = 3) (**h**’–**l’**) embryos from E10.5 to E13.5. The dynamic distribution of *LacZ*-positive cells in the craniofacial region of *COUP-TFII* ^*Z*/+^ embryos resembled that of *Wnt1-Cre*/+*;COUP-TFII* ^*flox*/+^ embryos. Initially, strongly *LacZ*-positive cells were differentially distributed within PA1 and PA2 in E10.5 embryos. As development proceeded, predominantly stained C-shaped rings (**l** and **l’**, arrows) rostral to the ear pinna were detected in E13.5 embryos. Higher magnifications of the ear region in **k** and **k’** are shown in **l** and **l’**, respectively. CF, cephalic flexure; di, diencephalon; e, eye; h, heart; hi, hindbrain; me, mesencephalon; tel, telencephalon. Scale bar = 1 mm in a and **h**–**l’**, 100 μm in **b**–**g**.

To explore whether COUP-TFII plays a physiological role in regulating development of the NCC lineage, we examined the spatiotemporal expression of COUP-TFII. First, we monitored COUP-TFII expression by performing whole-mount X-Gal staining of *COUP-TFII* ^*Z*/+^ embryos^31^, in which the *LacZ* reporter expression is driven by the *COUP-TFII* promoter. In E10.5 *COUP-TFII* ^*Z*/+^ embryos, X-Gal staining revealed relatively high levels of COUP-TFII expression in several embryonic regions, including the caudal telencephalon, posterior two-thirds of the diencephalon, diencephalon-mesencephalon boundary, eye, cephalic flexure, hindbrain, and craniofacial ganglia (Fig. 1a). This expression pattern recapitulates the expression profile of endogenous COUP-TFII^32,33^. We also observed moderate X-Gal-positive signals in pharyngeal arches (PAs), especially in the posterior part of PA1 and the anterior part of PA2 (Fig. 1a). Dual immunofluorescence staining of E10.5 *Wnt1-Cre*/+*;R26R* ^*mTmG/*+^ embryonic sections further showed COUP-TFII expression in the cephalic mesenchyme and pharyngeal endoderm as well as in the pharyngeal mesenchyme subpopulation (Fig. 1b and e). This COUP-TFII expression in GFP-positive mesenchymal cells of the PAs confirmed that COUP-TFII is expressed in specific tissues of NCC origin (Fig. 1b–g). Next, we performed whole-mount X-Gal staining of *COUP-TFII* ^*Z*/+^ embryos to further characterize the expression of COUP-TFII during craniofacial development. We observed spatiotemporal changes in the distribution of strongly X-Gal-stained cells as development proceeded (Fig. 1h–l), indicating that COUP-TFII exhibits a dynamic expression pattern during craniofacial morphogenesis. Also, we noticed that strongly X-Gal-stained cells located within PAs were gradually confined to the corner of the mouth and showed a C-shaped ring rostral to the ear pinna at E13.5 (Fig. 1l).

Next, to characterize the COUP-TFII expression profile in NCCs and their derivatives, we performed lineage-tracing experiments using *Wnt1-Cre*/+*;COUP-TFII* ^*flox*/+^ mice, in which *LacZ* reporter expression is under control of the *COUP-TFII* promoter upon Cre-mediated *COUP-TFII* deletion (Fig. 1h’–l’). The dynamic distribution of strongly X-Gal-stained cells in *Wnt1-Cre*/+*;COUP-TFII* ^*flox*/+^ embryos was comparable to that of *COUP-TFII* ^*Z*/+^ embryos from E10.5 to E13.5. These results show that craniofacial cells with high COUP-TFII expression mainly reside within the NCC lineage during development, and therefore COUP-TFII may play an important role in guiding the development of a specific NCC subpopulation.

### Loss of *COUP-TFII* leads to malformation of the tympanic ring and external acoustic meatus (EAM)

**Figure 2:**
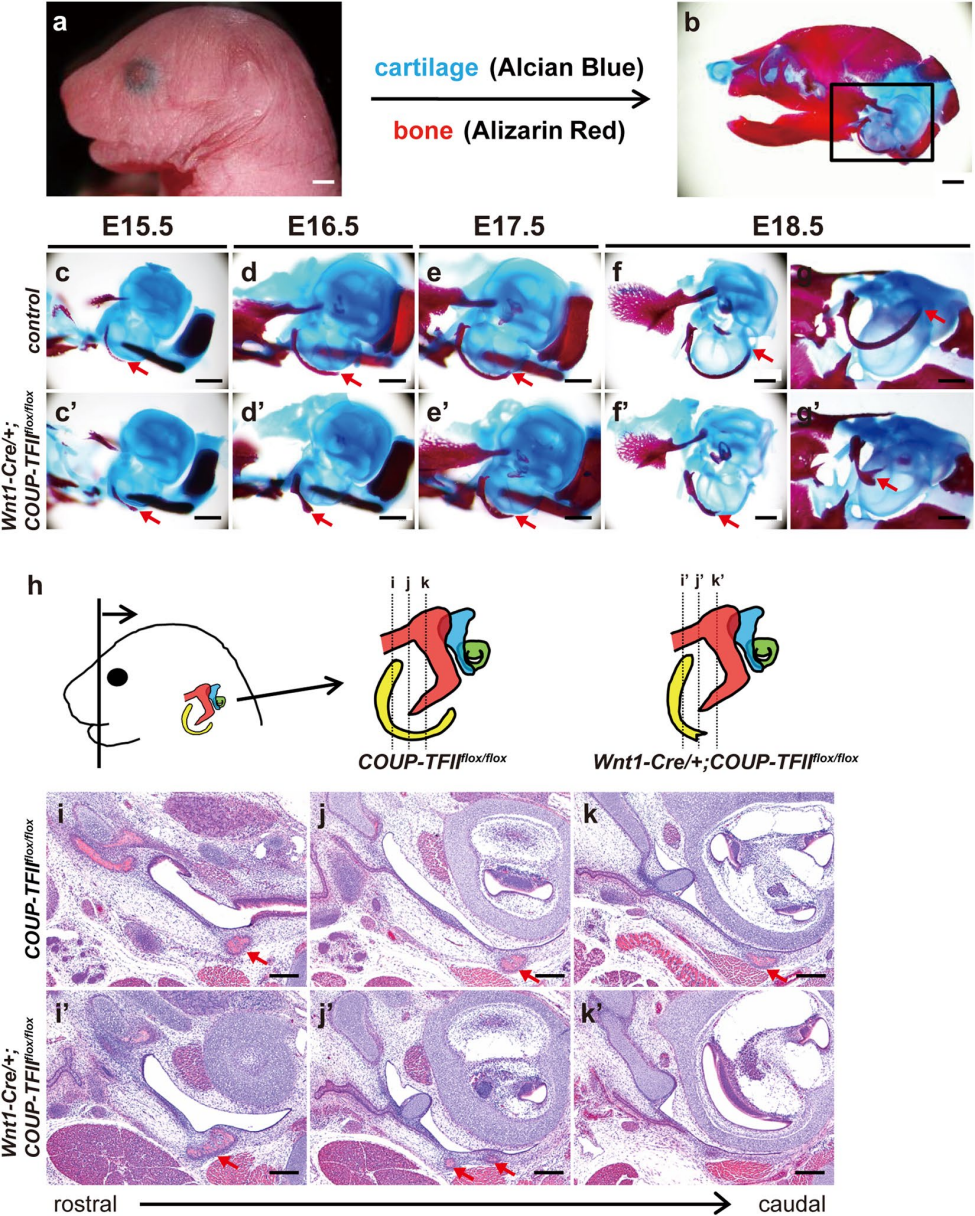
Malformation of the tympanic ring in *Wnt1-Cre*/+*;COUP-TFII* ^*flox/flox*^ mutants. (**a**) Gross appearance of E18.5 control mouse. (**b**) The skeletons of E18.5 control mice were stained with Alcian Blue and Alizarin Red to identify cartilages and bones, respectively. The tympanic ring is shown in the boxed area. (**c**–**g’**) Skeletal staining of littermate controls (E15.5, n = 3; E16.5, n = 8; E17.5, n = 4; E18.5, n = 13) (**c**–**g**) and *Wnt1-Cre*/+*;COUP-TFII* ^*flox/flox*^ mutants (E15.5, n = 3; E16.5, n = 3; E17.5, n = 3; E18.5, n = 11) (**c’**–**g’**) from E15.5 to E18.5. Tympanic ring development (indicated by arrow) proceeded normally in controls, whereas the tympanic ring was thickened and shortened in *Wnt1-Cre*/+*;COUP-TFII* ^*flox/flox*^ mutants. Inferior view of abnormal bifurcated tympanic ring in *Wnt1-Cre*/+*;COUP-TFII* ^*flox/flox*^ mutants (**g’**). (**h**–**k’)** Illustrations of the anatomical orientation (frontal section) and approximate planes of sections through the tympanic ring (depicted in yellow) from rostral to caudal^58^ (modified from ref.^58^) in controls and *Wnt1-Cre*/+*;COUP-TFII* ^*flox/flox*^ mutants Corresponding images of each indicated plane are shown in **i**–**k’**. A comparison of hematoxylin and eosin-stained serial sections confirmed that the tympanic ring (indicated by the arrow) in E18.5 *Wnt1-Cre*/+*;COUP-TFII* ^*flox/flox*^ mutants (n = 3) was markedly shorter than that in littermate controls (n = 4). A bifurcated end (**j’**, arrows) was also observed in *Wnt1-Cre*/+*;COUP-TFII* ^*flox/flox*^ mutants. Scale bar = 1 mm in a and b, 500 μm in **c**–**g’**, 200 μm in **i**–**m’**.

Cranial NCCs and their derivatives can differentiate not only into cranial ganglia and connective tissues but also into bone and cartilage in the head and neck^1^. Therefore, we stained cranial skeletal preparations with Alcian Blue and Alizarin Red to examine skull architecture (Fig. 2a–b). We observed that the tympanic ring, which develops to provide physical support for the tympanic membrane, was slightly thicker in E15.5 *Wnt1-Cre*/+*;COUP-TFII* ^*flox/flox*^ mutants (Fig. 2c’) than in littermate controls (Fig. 2c). From E16.5 onward, the normal C-shaped tympanic ring gradually developed in control embryos (Fig. 2d–g). In contrast, the tympanic ring in *Wnt1-Cre*/+*;COUP-TFII* ^*flox/flox*^ mutants was noticeably shortened. As development proceeded, abnormal bifurcation of the tympanic ring was also seen in *Wnt1-Cre*/+*;COUP-TFII* ^*flox/flox*^ mutants with full penetrance (Fig. 2d’–g’). Comparative histological analyses further confirmed the shortening and abnormal bifurcation of the tympanic ring in *Wnt1-Cre*/+*;COUP-TFII* ^*flox/flox*^ mutants (Fig. 2i–k’).

**Figure 3:**
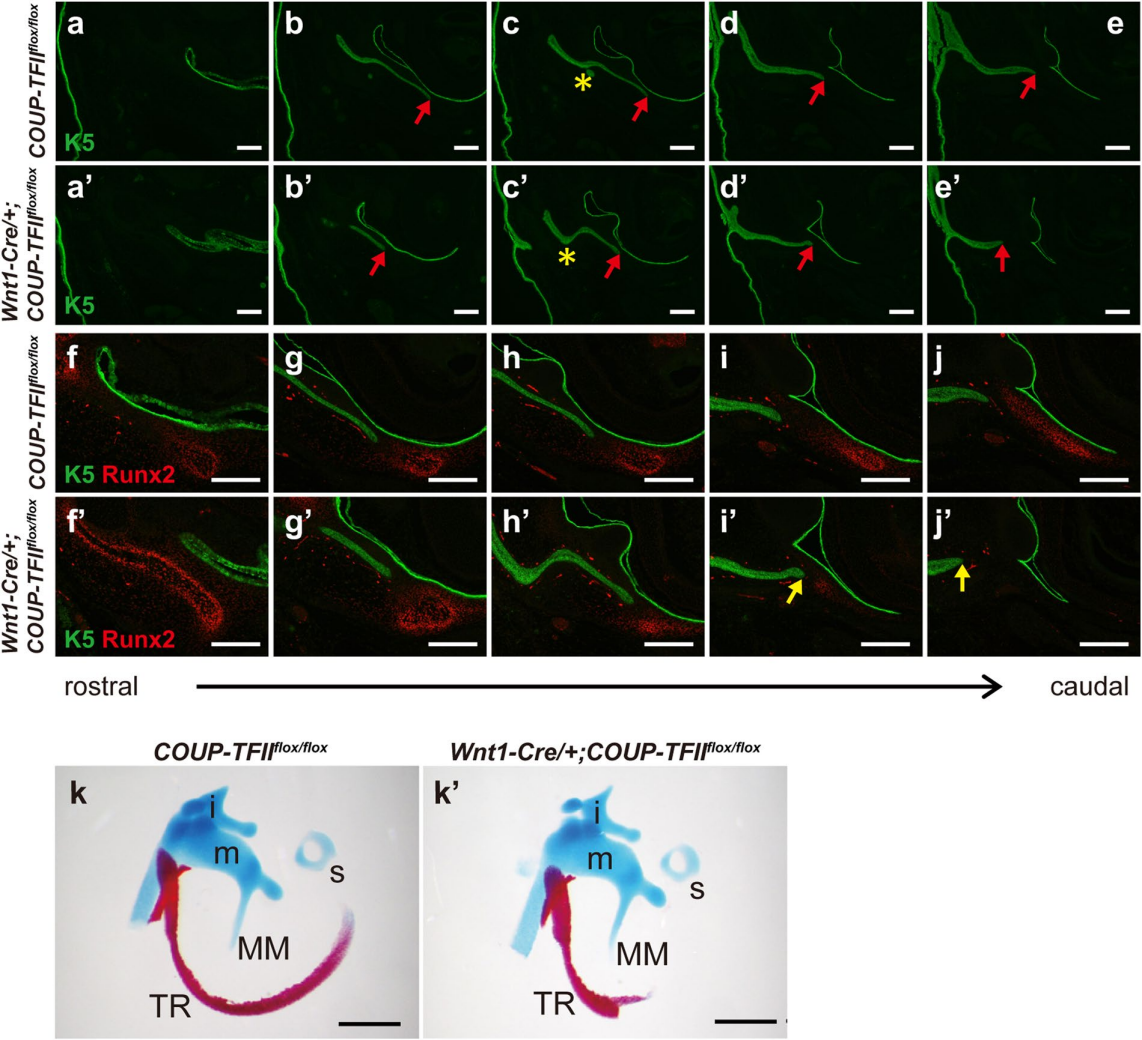
Abnormal EAM invagination does not perturb the development of the MM in *Wnt1-Cre*/+*;COUP-TFII* ^*flox/flox*^ mutants. (**a**–**j’**) Serial frontal head sections through the middle ear region from E16.5 controls (n = 5) (**a**–**j**) and *Wnt1-Cre*/+*;COUP-TFII* ^*flox/flox*^ mutants (n = 4) (**a’–j’**) were stained with the epithelial cell marker K5 (green) and osteogenic lineage marker Runx2 (red) to identify the spatial location of the EAM (red arrows) and tympanic ring, respectively. Compared with controls, *Wnt1-Cre*/+*;COUP-TFII* ^*flox/flox*^ mutants showed defective morphogenesis of EAM invagination (yellow asterisk in **c** and **c’**). In both control and mutant mice, in the presence of the tympanic ring, the extremity of the EAM pointed to the position of the tympanic ring. However, the tympanic ring ended rostrally so that the direction of EAM invagination was misaligned in the caudal region (**i’** and **j’**, yellow arrow) of *Wnt1-Cre*/+*;COUP-TFII* ^*flox/flox*^ mutants. (**k–k’**) Alcian Blue and Alizarin Red-stained middle ear. MM structure was comparable between controls (n = 3) and *Wnt1-Cre*/+*;COUP-TFII* ^*flox/flox*^ mutants (n = 3). TR, tympanic ring; **i**, incus; **m**, malleus; MM, manubrium of the malleus; s, stapes. Scale bar = 200 μm in **a**–**j’**, 500 μm in **k** and **k’**.

The tympanic ring directs the invagination of the EAM, which later forms the external ear canal^34^. The EAM is an ectodermal epithelial cell layer that directly originates from the first pharyngeal cleft. Therefore, we utilized the epithelial cell marker cytokeratin 5 (K5) and osteogenic lineage marker Runx2 to designate the spatial locations of the EAM and tympanic ring, respectively. Dual immunofluorescence analysis of K5 and Runx2 revealed defective morphogenesis of the EAM in *COUP-TFII* mutants (Fig. 3a–j’). The configuration of EAM invagination in *Wnt1-Cre*/+*;COUP-TFII* ^*flox/flox*^ mutants was different from that of littermate controls (Fig. 3c and c’). In the presence of the tympanic ring, the extremity of EAM invagination was directed toward the position of the tympanic ring in both control and mutant embryos (Fig. 3f–j and f’–h’). However, in the caudal part, *Wnt1-Cre*/+*;COUP-TFII* ^*flox/flox*^ mutants exhibited a misalignment of EAM invagination (Fig. 3i’ and j’), which was a likely consequence of the premature ending and bifurcation of the mutant tympanic ring. Proper EAM invagination is crucial for coordinating the development and positioning of the manubrium of the malleus (MM)^34^. Therefore, it was surprising that the anomalies in the tympanic ring and EAM invagination of *Wnt1-Cre*/+*;COUP-TFII* ^*flox/flox*^ mutants did not perturb the coordinated development of the MM (compare Fig. 3k’ with k), indicating that the rostral half of the tympanic ring is sufficient to guide MM formation.

### Loss of *COUP-TFII* up-regulates Sox9 in the tympanic ring primordium

**Figure 4:**
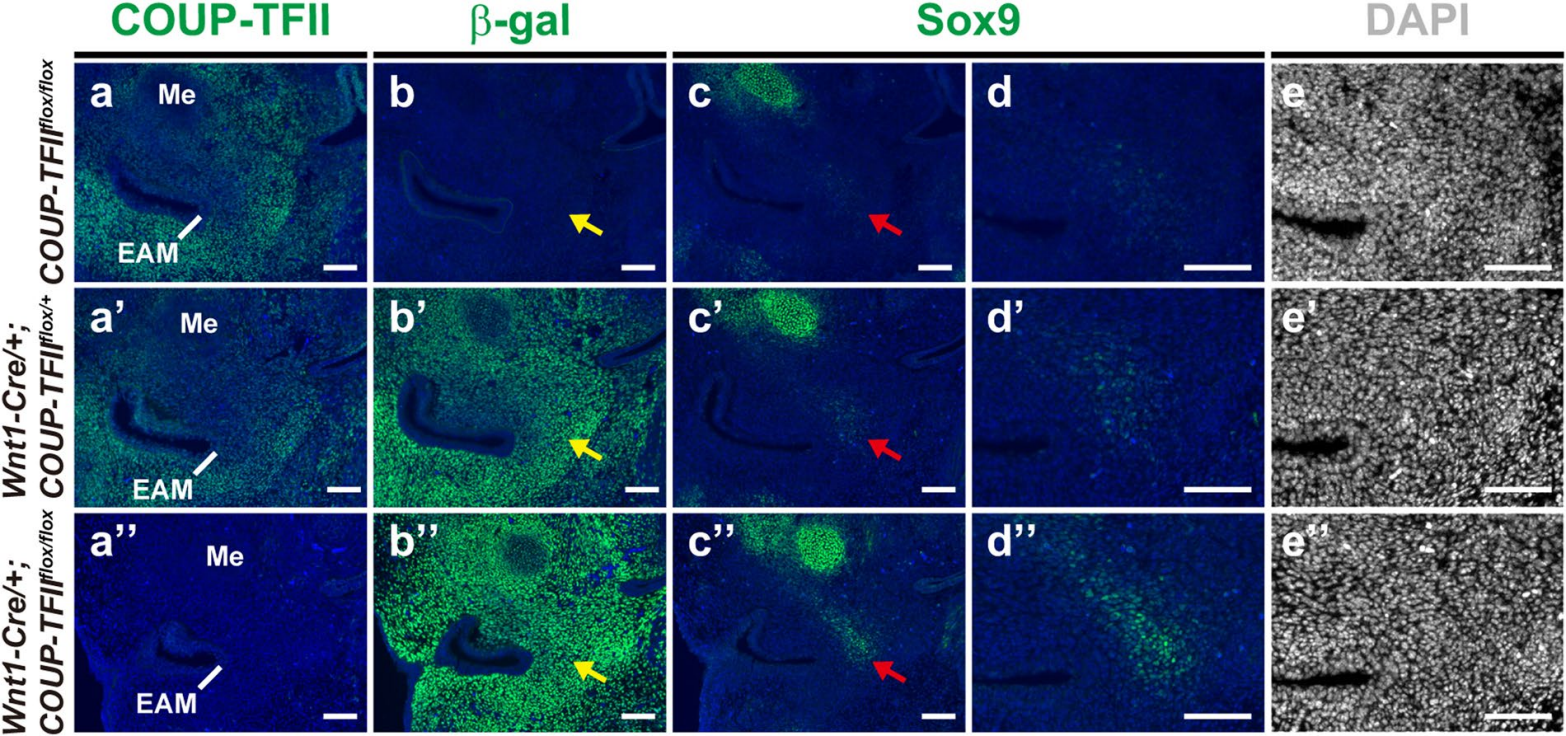
Up-regulation of Sox9 precedes mesenchymal condensation in the presumptive tympanic ring primordium of E13.5 *Wnt1-Cre*/+*;COUP-TFII* ^*flox/flox*^ mutants. Adjacent frontal sections through the otic region from early E13.5 embryos were immunostained with antibodies against COUP-TFII, β-gal, and Sox9. (**a**–**a”**) COUP-TFII was detected broadly in cranial mesenchyme around the EAM in *COUP-TFII* ^*flox/flox*^ controls (n = 4). COUP-TFII expression was moderately decreased and undetectable in *Wnt1-Cre*/+*;COUP-TFII* ^*flox*/+^ (n = 5) (**a’**) and *Wnt1-Cre*/+*;COUP-TFII* ^*flox/flox*^ (n = 5) (**a”**) embryos, respectively, compared with control embryos. (**b**–**b”**) The β-gal signal in cranial mesenchyme around the EAM was inversely correlated with COUP-TFII expression in each genotype. The spatial distribution of β-gal-positive cells was indistinguishable between *Wnt1-Cre*/+*;COUP-TFII* ^*flox*/+^ (n = 8) and *Wnt1-Cre*/+*;COUP-TFII* ^*flox/flox*^ mice (n = 8). There is no β-gal signal in *COUP-TFII* ^*flox/flox*^ controls (n = 6). (**c**–**d”**) Sox9 was weakly expressed in a subpopulation of mesenchymal cells (indicated by red arrow) medial to the EAM in *COUP-TFII* ^*flox/flox*^ controls (n = 8) (**c**). The intensity of Sox9 expression was slightly increased in *Wnt1-Cre*/+*;COUP-TFII* ^*flox*/+^ controls (n = 11) (**c’**), whereas Sox9 expression was dramatically up-regulated in *Wnt1-Cre*/+*;COUP-TFII* ^*flox/flox*^ mutants (n = 11) **(c”)**. Sox9 was also detected in the corresponding β-gal-expressing region. The corresponding Sox9-expressing area is indicated by the yellow arrow in **b**–**b”**. Higher magnifications of the Sox9-expressing area in **c**–**c”** are shown in **d**–**d”**, respectively. (**e**–**e”**) All these Sox9-expressing cells in **d**–**d”** were evenly distributed at this stage (n = 4). Nuclei were stained with DAPI (blue in **a**–**d”** and grey in **e**–**e”**). EAM, external acoustic meatus; Me, Meckle’s cartilage. Scale bar = 100 μm.

Sox9 expression is progressively down-regulated in the osteogenic cell compartment during intramembranous ossification, indicating that Sox9 may be involved in incipient intramembranous bone formation^35,36^. However, the role of Sox9 in the development of intramembranous bones remains poorly understood. In mice, the primordium for the tympanic ring becomes apparent by E13.5 as a condensation ventral to the first pharyngeal cleft lateral to Meckel’s cartilage^37^. To investigate whether Sox9 contributes to malformation of the tympanic ring in *Wnt1-Cre*/+*;COUP-TFII* ^*flox/flox*^ mutants, we first performed COUP-TFII, β-galactosidase (β-gal), and Sox9 immunostaining of E13.5 head sections. COUP-TFII was detected in almost all cranial mesenchyme but not in the EAM around the otic region of *COUP-TFII* ^*flox/flox*^ control mice. Compared with *COUP-TFII* ^*flox/flox*^ control embryos (Fig. 4a), COUP-TFII levels were moderately decreased and nearly undetectable in *Wnt1-Cre*/+*;COUP-TFII* ^*flox*/+^ (Fig. 4a’) and *Wnt1-Cre*/+*;COUP-TFII* ^*flox/flox*^ (Fig. 4a”) embryos, respectively. The profiles of Wnt1-Cre-mediated *COUP-TFII* ablation, as visualized by anti-β-gal staining in cranial mesenchymes around the EAM, were indistinguishable between *Wnt1-Cre*/+*;COUP-TFII* ^*flox*/+^ (Fig. 4b’) and *Wnt1-Cre*/+*;COUP-TFII* ^*flox/flox*^ (Fig. 4b”) embryos except that the intensity of β-gal staining (Fig. 4b–b”) was inversely correlated with levels of COUP-TFII. Interestingly, up-regulation of Sox9 was observed in the specific subset of mesenchymal cells medial to the EAM and lateral to Meckel’s cartilage, where the future tympanic ring is situated, in E13.5 *Wnt1-Cre*/+*;COUP-TFII* ^*flox*/+^ (Fig. 4c’) and *Wnt1-Cre*/+*;COUP-TFII* ^*flox/flox*^ (Fig. 4c”) embryos. Moreover, we detected Sox9 up-regulation in the corresponding β-gal-expressing region. Notably, no obvious cellular condensation was observed in this Sox9-expressing population at this stage (Fig. 4e–e”). To ensure equivalent fluorescent intensity of β-gal-expressing cells in control and mutant embryos, *Wnt1-Cre*/+*;COUP-TFII* ^*flox*/+^ mice were mated with *COUP-TFII* ^*+/−*^ mice to generate *Wnt1-Cre*/+*;COUP-TFII* ^*flox*/+^ and *Wnt1-Cre*/+*;COUP-TFII* ^*flox/−*^ mice that contained only one *LacZ* allele. Additional immunohistochemical analyses showed comparable expression patterns of β-gal between *Wnt1-Cre*/+*;COUP-TFII* ^*flox*/+^ and *Wnt1-Cre*/+*;COUP-TFII* ^*flox/−*^ embryos (Supplementary Fig. S1b-b”). We also confirmed that Sox9 expression was up-regulated in the subpopulation of evenly distributed β-gal-positive mesenchyme in *Wnt1-Cre*/+*;COUP-TFII* ^*flox/−*^ mutants (Supplementary Fig. S1). These results indicate that loss of *COUP-TFII* does not interrupt the migration and localization of NC lineage cells to the otic region but results in Sox9 up-regulation in the subset of COUP-TFII-expressing NCC-derived mesenchyme that may give rise to the tympanic ring before mesenchymal condensation. The differential expression of Sox9 in tympanic ring became evident at E14.5 (Supplementary Fig. S2a-e’). We observed consistent up-regulation of Sox9 in *Wnt1-Cre*/+*;COUP-TFII* ^*flox/flox*^ mutants compared with control mice, and those Sox9-expressing mesenchymal cells in mutants also exhibited abnormal expansion with a bifurcated end (compare Supplementary Fig. S2a’–b’ with a–b). In control embryo, Sox9-expressing mesenchymal cells were detected in all examined otic region. However, in *Wnt1-Cre*/+*;COUP-TFII* ^*flox/flox*^ mutants, Sox9-expressing mesenchymal cells were not seen in the caudalmost part of examined region (Supplementary Fig. S2e’ and e). Consistent with the previous study showing that COUP-TFII expression is gradually down-regulated during osteoblast differentiation^38^, we noticed that there was a much weaker β-gal signal in the corresponding Sox9-expressing region at E14.5 (Supplementary Fig. S2f–j’), which is in contrast to the uniform β-gal expression in cranial mesenchyme around the E13.5 otic region. These results further suggest that Sox9 is expressed in the primordium for the future tympanic ring.

**Figure 5:**
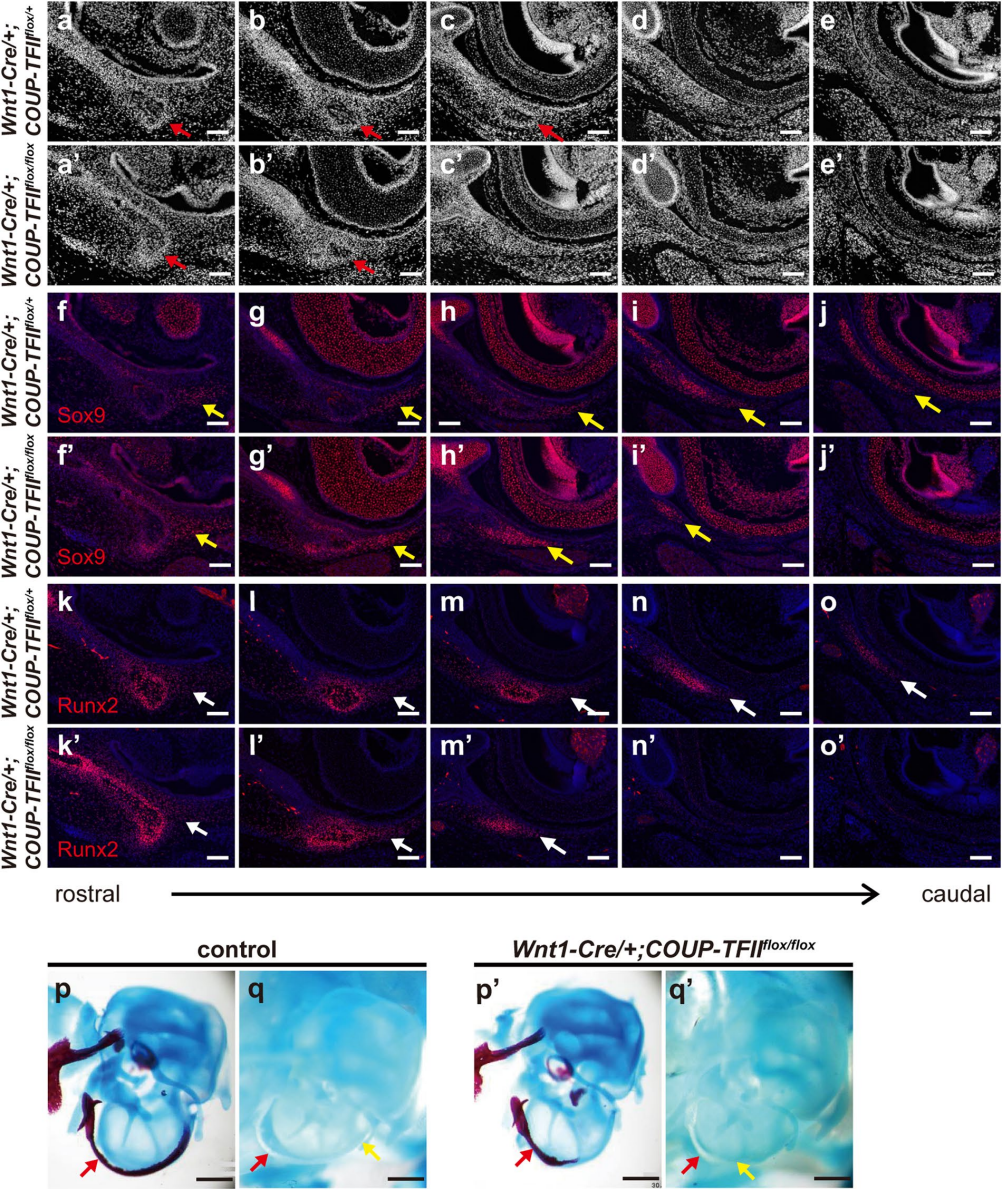
Aberrant Sox9 up-regulation in the osteogenic mesenchyme does not lead to the formation of ectopic cartilage in the tympanic ring of *Wnt1-Cre*/+*;COUP-TFII* ^*flox/flox*^ mutants. (**a**–**o’**) Adjacent frontal sections from E16.5 *Wnt1-Cre*/+*;COUP-TFII* ^*flox*/+^ controls and *Wnt1-Cre*/+*;COUP-TFII* ^*flox/flox*^ mutants were immunostained with anti-Sox9 and anti-Runx2 antibodies and counterstained with DAPI. Tissue architecture around the otic region is visualized by DAPI (gray) staining (**a**–**e’**). At E16.5, the bony structure of the tympanic ring (indicated by red arrow) can be clearly identified from surrounding mesenchyme. Mesenchymal Sox9 expression (red, indicated by yellow arrow) was persistently up-regulated in E16.5 *Wnt1-Cre*/+*;COUP-TFII* ^*flox/flox*^ mutants (n = 5) (**f’–j’**), and its spatial distribution was also different from that of controls (n = 6) (**f**–**j**). Runx2 was expressed in both the bony part and the surrounding mesenchyme of the tympanic ring (**k**–**o’**). Histological analysis revealed that Sox9 expression in the surrounding mesenchyme was localized to the corresponding lower Runx2-expressing osteogenic cells (white arrow) in both control and mutant embryos (n = 2). (**p–q’**) Lateral view of E18.5 middle ear. Alcian Blue and Alizarin Red staining of the middle ear revealed that the ossified tympanic ring was shorter in *Wnt1-Cre*/+*;COUP-TFII* ^*flox/flox*^ mutants (n = 13) (**p’**) compared with controls (n = 11) **(p**). Single Alcian Blue staining showed no cartilage formation in the tympanic ring of controls (n = 8) (q) or *Wnt1-Cre*/+*;COUP-TFII* ^*flox/flox*^ mutants (n = 5) (**q’**). The red arrow denotes the position of the ossified tympanic ring, and the yellow arrow indicates the endpoint of the tympanic ring. Scale bar = 100 μm in **a**–**j’**, 500 μm in **k**–**l’**.

At a later stage (E16.5) when the bony structure of the tympanic ring can be clearly identified by histological analysis, the level of Sox9 expression was lower in the bony part of the tympanic ring than in the surrounding mesenchyme in both control and mutant embryos (Fig. 5a–j’), consistent with previous studies reporting the down-regulation of Sox9 expression during intramembranous ossification^35,36^. We also noticed that high levels of Sox9 were detected in the mesenchyme around the caudal end of developing tympanic ring in both control and *COUP-TFII* mutant embryos (control – Fig. 5i and j; mutant – Fig. 5h’ and i’). Nevertheless, the Sox9 expression in *Wnt1-Cre*/+*;COUP-TFII* ^*flox/flox*^ mutants was consistently higher in the surrounding mesenchyme of the rostral part of tympanic ring than that of the control mice (compare Fig. 5f–h with f’–g’). The master regulator of osteogenesis, Runx2, was highly expressed in the bony part of the tympanic ring, and more moderate levels were observed in Sox9-positive surrounding mesenchymal cells in both control and *Wnt1-Cre*/+*;COUP-TFII* ^*flox/flox*^ mutant mice (Fig. 5k–o’). These results suggest that COUP-TFII may coordinate the tympanic ring development by inhibiting Sox9 expression in the osteogenic mesenchyme. Given that Sox9 is required for determining chondrogenic cell lineage and is sufficient for cartilage formation^8,14,39,40^, we next investigated whether malformations of the tympanic ring in *Wnt1-Cre*/+*;COUP-TFII* ^*flox/flox*^ mutants result from inaccurate cell fate decision (i.e., chondrogenic cell fate) due to abnormal up-regulation of Sox9. However, skeletal preparations revealed that corresponding regions of the Alizarin Red-stained tympanic ring showed no Alcian Blue (i.e., cartilage) staining in either E18.5 controls or *Wnt1-Cre*/+*;COUP-TFII* ^*flox/flox*^ mutants (Fig. 5p–q’). Thus, aberrant Sox9 up-regulation did not result in the diversion of osteogenic mesenchymal cells toward cartilage differentiation. Collectively, our findings indicate that *COUP-TFII*-null mesenchymal cells are able to condense, differentiate into osteoblasts, and form a truncated tympanic ring in *Wnt1-Cre*/+*;COUP-TFII* ^*flox/flox*^ mutants through intramembranous ossification.

### COUP-TFII governs the distribution of preosteoblasts during tympanic ring development

**Figure 6:**
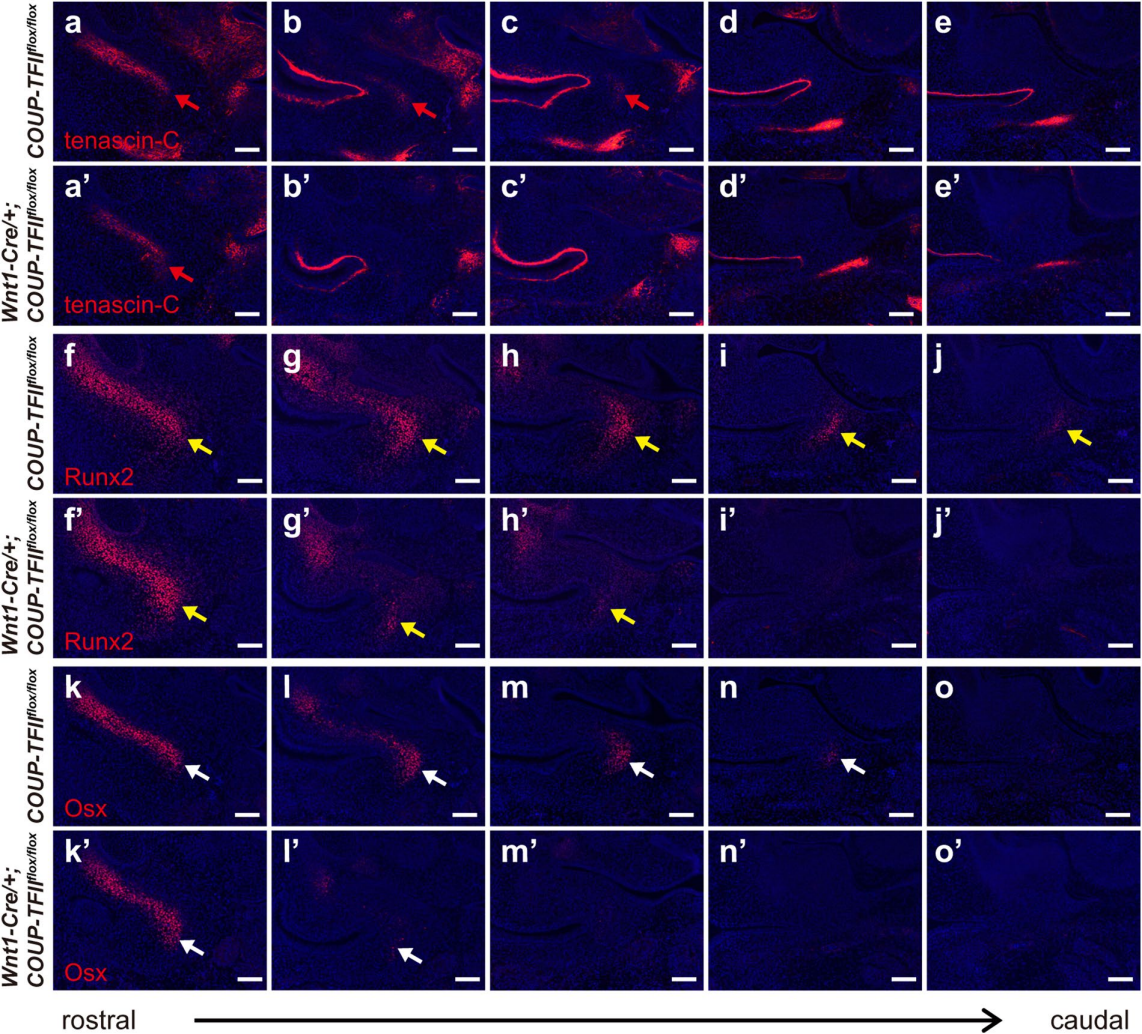
The distribution of preosteoblasts is rostrally restricted in the ear region of E13.5 *Wnt1-Cre*/+*;COUP-TFII* ^*flox/flox*^ mutants. Adjacent frontal sections, through the otic region from late E13.5 embryos (n = 5 for control and n = 3 for mutant), were immunostained with antibodies against tenascin-C, Runx2, and Osx. (**a**–**e’**) At late E13.5, condensed mesenchymal cells in the rostral part of the tympanic ring primordium exhibited tenascin-C expression (indicated by red arrow) in controls (**a**–**e**) and *Wnt1-Cre*/+*;COUP-TFII* ^*flox/flox*^ mutants (**a’–e’**). However, examination of the serial sections through the otic region revealed that tenascin-C-expressing condensed mesenchyme was detected in the first three sections (**a**–**c**) of control mice, while that was detected only in the rostralmost section (**a’**) of *Wnt1-Cre*/+*;COUP-TFII* ^*flox/flox*^ mutants. (**f**–**o’**) Both Runx2- (**f**–**j’**, indicated by yellow arrow) and Osx-expressing (**k**–**o’**, indicated by white arrow) mesenchymal cells were not detected in the caudal region of *Wnt1-Cre* ^/+^ *;COUP-TFII* ^*flox/flox*^ mutants, while such cells could easily be seen in the counterpart of control mice. In addition to condensed mesenchyme, Runx2 and Osx were also expressed in non-condensed mesenchyme in the caudal part of the tympanic ring primordium, which showed no tenascin-C expression. Scale bar = 100 μm.

Mesenchymal condensation is a crucial stage for skeletal patterning during bone development. Therefore, we analyzed mesenchymal condensation to verify whether the *COUP-TFII*-null mesenchyme exhibits impaired skeletal patterning for the future tympanic ring. Tenascin-C, a glycoprotein of extracellular matrix components, is involved in establishing the condensation boundary of the osteogenic population from non-skeletogenic mesenchymes^41–43^. At E13.5, tenascin-C expression was detected in the condensed mesenchyme as evidenced by cell aggregation but not in the non-condensed mesenchyme of the developing tympanic ring in both control and mutant embryos (Fig. 6a–e’). Loss of *COUP-TFII* in NCCs did not affect the level of tenascin-C expression, but tenascin-C-positive condensed mesenchyme within the developing tympanic ring of *Wnt1-Cre*/+*;COUP-TFII* ^*flox/flox*^ mutants did not extend caudally as far as the condensed mesenchyme did in control mice (compare Fig. 6c’ with c). This result revealed that the abnormal shortening of condensed mesenchymal structure contributes to anomalies of the tympanic ring in *Wnt1-Cre*/+*;COUP-TFII* ^*flox/flox*^ mutants. Similarly, expression of the osteogenic markers Runx2 and Osterix (Osx) at the single-cell level were comparable between the corresponding regions within the developing tympanic rings of control and *COUP-TFII* mutant embryos. However, both Runx2- and Osx-expressing mesenchymal cells were not detected in the caudal region of *Wnt1-Cre*/+*;COUP-TFII* ^*flox/flox*^ mutants, while such cells could easily be seen in the counterpart of control mice (Fig. 6f–o’). According to previous studies, specified preosteoblasts express Runx2, and at a later stage, preosteoblast committed to an osteoblast lineage express both Runx2 and Osx^44^. Therefore, these results revealed that, instead of extending into more caudal region as in control mice, the distributions of both specified and committed preostroblasts were rostrally restricted in *Wnt1-Cre*/+*;COUP-TFII* ^*flox/flox*^ mutants. Interestingly, we noted that Runx2- and Osx-positive signals were detected not only in condensed mesenchyme but also in non-condensed mesenchyme at the caudal part of the developing tympanic ring (Fig. 6a–o’). Thus, the distributions of Runx2- and Osx-expressing cells were much broader than that of tenascin-C-expressing cells, which indicates that, before mesenchymal condensation, these Runx2- and Osx-expressing preosteoblasts already exist during tympanic ring development. Taken together, our results suggest that loss of *COUP-TFII* results in aberrant up-regulation of Sox9 in the subpopulation of NCC-derived mesenchyme and affects the distribution of preosteoblasts, which in turn leads to improper pattern of mesenchymal condensation and consequently truncated tympanic ring formation.

## Discussion

The work presented here unveils the differential expression of COUP-TFII and its biological function in cranial NCCs and their derivatives *in vivo*. We showed that targeted inactivation of *COUP-TFII* in the NCC lineage leads to tympanic ring abnormalities. Genetic evidence showed that *COUP-TFII* deficiency results in the aberrant expression/regulation of Sox9 in the primordium of the tympanic ring and affects the distribution of preosteoblasts before mesenchymal condensation.

*In vivo* fate mapping and comparative histological analyses showed that loss of *COUP-TFII* affects neither NCC migration from neural folds to the ear region nor survival during this period. Before mesenchymal condensation, we detected COUP-TFII loss-of-function and increased Sox9 protein levels in the mesenchyme that presumably gives rise to the tympanic ring in E13.5 *Wnt1-Cre*/+*;COUP-TFII* ^*flox/flox*^ mutants, suggesting that COUP-TFII may negatively regulate Sox9 expression in the osteogenic mesenchyme. This result stands in contrast to the previous finding showing that COUP-TFII is recruited to the Sox9 regulatory region by Sp1 to activate the expression of Sox9, leading to the chondrogenic commitment of mesenchymal precursors^27^. The underlying basis for the inverse relationship between COUP-TFII and Sox9 during osteogenesis versus chondrogenesis is currently not clear. One possibility is that COUP-TFII interacts with other transcription factors to cooperatively inhibit Sox9 expression, whereas another possibility is that Sox9 is an indirect target of COUP-TFII-mediated repression. Future studies are needed to delineate how COUP-TFII regulates Sox9 expression in osteogenesis. In addition, COUP-TFII physically interacts with Runx2 and interferes with its binding on the target promoter, which inhibits the transcriptional activity, but not the level of Runx2, in directed osteoblast differentiation^27,38^. However, we found that loss of *COUP-TFII* does not affect the expression level of Runx2 or its direct targets, Osx and osteocalcin (OC) (Supplementary Fig. S3), suggesting that COUP-TFII does not regulate Runx2 transactivity during osteoblast differentiation in the developing tympanic ring.

We detected Runx2- and Osx-positive signals, which indicate commitment of preosteoblasts to an osteoblast lineage, in dispersed mesenchyme, as well as in condensed mesenchyme (i.e., tenascin-C-expressing cells or the cell aggregation in tissues), in the developing tympanic ring, which reveals that preosteoblast commitment precedes mesenchymal condensation during tympanic ring development. A similar process has been observed during the development of the chicken mandible^45^, which also occurs through intramembranous ossification. Therefore, our findings provide further evidence supporting that osteogenesis is different from chondrogenesis, in which mesenchymal condensation triggers prechondroblast commitment^46,47^.

In skeletal development, Sox9 and Runx2 are the major transcription factors for chondrogenesis and osteogenesis, respectively. The spatiotemporal overlap of Sox9 and Runx2 expression unveils lineage-specific roles in orchestrating transcription of downstream targets during bone formation. Loss of Sox9 in NCCs and growth plate result in ectopic expression of osteoblast marker genes, such as *Runx2*, *Osx* and *Col1a1*, in the presumptive cartilage existing in wild-type embryos and differentiated growth plate chondrocytes, respectively^8,9^. In addition to directly activating the expression of major cartilage-specific extracellular matrix genes, Sox9 is also capable of suppressing Runx2 expression to specify and maintain the chondrogenic lineage fate^48–50^. By contrast, the role of Sox9 in osteogenesis is not well understood. Sox9 expression is gradually down-regulated during intramembranous bone formation^35,36^. In cranial skeletogenesis, misexpression of Sox9 results in down-regulation of *Runx2* in chondrocytes of the ceratobranchial cartilage, whereas the expression of Runx2 in osteoblasts and osteogenic mesenchymal condensation are not disrupted in the intramembranous surangular bone^14^. Further mechanistic investigation showed that Sox9 suppresses the transactivity of Runx2 in the established osteoblast lineage, which hinders osteoblast differentiation^51^. Our study provides additional *in vivo* evidence that the regulation of Runx2 by Sox9 during osteogenesis is different from that during chondrogenesis. Despite the shortened primordium of the tympanic ring in *Wnt1-Cre*/+*;COUP-TFII* ^*flox/flox*^ mutants, abnormal Sox9 up-regulation did not affect the expression of Runx2, and Runx2-positive cells still resided within their anatomical locations, consistent with previous observations in surangular bone^14^. Notably, the expression levels of Osx and OC, the direct targets of Runx2, were not altered, indicating that abnormally increased Sox9 in the *Wnt1-Cre*/+*;COUP-TFII* ^*flox/flox*^ tympanic ring primordium does not inhibit the transactivity of Runx2. However, we did not rule out the possibility that the level of Sox9 might not have been sufficient to inhibit Runx2 transactivity in mutants. The inconsistencies among studies may also be due to different experimental approaches under varying physiological and cellular contexts.

Morphogenesis of the tympanic ring, EAM, and MM are spatially and temporally associated^37,52^. This close relationship is also observed through clinical experience with humans^53,54^. Analyses of genetically modified (*Gsc* ^*−/−*^ and *Prx1* ^*−/−*^ mutants) and retinoic acid-treated mouse embryos support the deduction that the tympanic ring is essential for inducing the invagination of the first pharyngeal cleft to form the EAM, which then provides signals that act on the underling mesenchymal cells to coordinate proper MM development^34,52,55–58^. It is noteworthy that the severity of tympanic ring anomaly is tightly associated with the level of MM deformity. Concurrent disappearance of the tympanic ring and MM is also found in endothelin A receptor-deficient mice^59,60^. Both *Gas1*-deficient and *Tshz1-*deficient mice have shorter and thicker tympanic rings accompanied by the absence of a MM^61,62^. *Bapx1* ^*−/−*^ embryos show relatively normal MM formation but exhibit hypoplasia (i.e., thinness) of the tympanic ring only in the anterior/rostral part^63^. Although EAM development has not been clearly described in every aforementioned mutant, studies consistently indicate that the MM forms in a coordinated manner alongside tympanic ring development. In this study, we found that the loss of *COUP-TFII* in the NCC lineage results in a shortened and thicker tympanic ring. Although this truncated tympanic ring was able to induce and guide the morphogenesis of EAM invagination, the extremity of caudal EAM invagination went astray. However, the MM was well developed and properly positioned in *Wnt1-Cre*/+*;COUP-TFII* ^*flox/flox*^ mutants, suggesting that the rostral half of the tympanic ring—rather than the entire structure—is sufficient to coordinate the development and positioning of the MM. Nevertheless, this hypothesis is inconsistent with phenotypes observed in *Tshz1* ^*−/−*^ mutants. The morphologies of the shortened tympanic rings in *Wnt1-Cre*/+*;COUP-TFII* ^*flox/flox*^ and *Tshz1* ^*−/−*^ mutants are similar to each other and to that of the rostral half of the control tympanic ring; however, MM development differs between these two mutants. Coré *et al*. showed that *Tshz1* is expressed in the mesenchyme surrounding the malleal primordium, implying that Tshz1 may play a cell-autonomous role in MM development. By contrast, down-regulation of COUP-TFII in the Sox9-positive developing malleus and neighboring mesenchyme was noted in control embryos from E12.5 (Supplementary Fig. S4). Therefore, the discrepancy in MM development between *Wnt1-Cre*/+*;COUP-TFII* ^*flox/flox*^ and *Tshz1* ^*−/−*^ mutants could result from a difference between the cell-autonomous roles of COUP-TFII and Tshz1.

In summary, our study establishes that COUP-TFII is an essential transcription factor in NCCs and their derivatives for tympanic ring development *in vivo*. Our results provide new insights into the differential roles of COUP-TFII in fine-tuning the expression of Sox9 and regulating the distribution of preosteoblasts at the beginning of intramembranous bone development. As previous studies report that COUP-TFII can specify the lineage commitment of mesenchymal stem/stromal cells (MSCs), prospective studies aimed at understanding the precise spatiotemporal control and plasticity of MSCs *in vivo* will be of great benefit to future MSCs-based therapy.

## Materials and Methods

### Mice

*COUP-TFII* ^*+/−*^^19^, *COUP-TFII* ^*flox/flox*^, and *COUP-TFII* ^*Z*/+^ ^31^ mice were kindly provided by Dr. Sophia Y. Tsai, Baylor College of Medicine, Houston, Texas. To inactivate *COUP-TFII* expression in the NCC lineage, *Wnt1-Cre* transgenic mice (Tg(*Wnt1-Cre*)11Rth)^30^ were crossed with *COUP-TFII* ^*flox/flox*^ mice. Double-heterozygous *Wnt1-Cre*/+*;COUP-TFII* ^*flox*/+^ mice were then mated with *COUP-TFII* ^*flox/flox*^ or *COUP-TFII* ^*+/−*^ mice to generate *Wnt1-Cre*/+*;COUP-TFII* ^*flox/flox*^ or *Wnt1-Cre*/+*;COUP-TFII* ^*flox/−*^ mice, respectively. For lineage-tracing experiments, the Cre reporter mouse line *R26R* ^*mTmG/+*^ ^64^ was crossed with *Wnt1-Cre* transgenic mice to characterize the distribution of neural crest derivatives as described previously^65^. The animal experiments were approved by the Institutional Animal Care and Use Committee (IACUC) of National Yang-Ming University, Taiwan. The animal care and experimental procedures were performed in accordance with the Guidelines of the IACUC of National Yang-Ming University, Taiwan.

### Whole-mount X-Gal staining

Embryos from timed-pregnant mice were collected and fixed in 2% paraformaldehyde (PFA; Electron Microscopy Science)/1 × phosphate-buffered saline (PBS), pH 7.3 for 10–15 min at 4 °C. Embryos were then incubated in X-Gal staining solution at 30 °C until appropriate color development and post-fixed in 4% PFA/PBS at 4 °C overnight. Detailed materials and procedures were described previously^24^.

### Histological and immunohistochemical staining

Histological and immunohistochemical experiments were performed as described previously^24,66^. Antibodies against β-gal (Chemicon, AB1211, 1:1200), COUP-TFII (Perseus Proteomics, PP-H7147, 1:1000), GFP (Genetex, GTX13970, 1:100), K5 (Abcam, ab24647, 1:125), Osx (Abcam, ab22552, 1:3000), Runx2 (Santa Cruz Biotechnology, sc10758 [M-70], 1:1000), Sox9 (Santa Cruz Biotechnology, sc20095 [H-90], 1:150), and tenascin-C (Millipore, AB19013, 1:3000) were used.

### Skeletal preparations

Skeletal staining was performed as described previously^65^ with slight modifications. Briefly, embryo heads were carefully skin-peeled and fixed in 95% ethanol followed by degreasing with acetone. Cartilage and bone were visualized by staining with 0.05% Alcian Blue 8GX (Sigma-Aldrich) in acid ethanol and 0.01% Alizarin Red S (Alfa Aesar) in 1% KOH, respectively. Soft tissues were cleared by 1% KOH. For single Alcian Blue staining, the step for Alizarin Red S staining was omitted, and the samples were directly immersed in 1% KOH.

## Electronic supplementary material

supplementary information
