# Supplementary material for: COUP-TFII is required for morphogenesis of the neural crest-derived tympanic ring
Source: Sci Rep. 2017 Sep 28;7:12386. doi: 10.1038/s41598-017-12665-0 (PMC5620064; doi:10.1038/s41598-017-12665-0)
Supplement: Supplementary file 1 — supplementary information [file 41598_2017_12665_MOESM1_ESM.pdf]

Supplementary Information

COUP-TFII is required for morphogenesis of the neural crest-derived tympanic ring

Wen-Hsin Hsu, Chun-Ming Chen, and Li-Ru You

Supplementary Materials and Methods

Immunohistochemical staining for osteocalcin (OC)

Antibody against OC (abcam, ab93876, 1:2000) was used, and the experiment procedure was performed as described in Materials and Methods.

Supplementary Figures

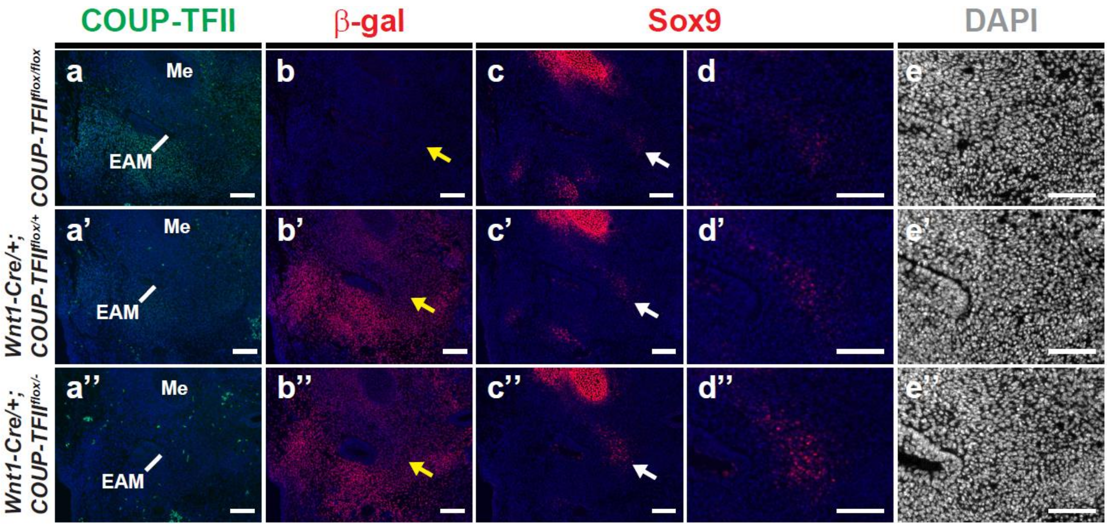

Fig. S1. Sox9 up-regulation precedes mesenchymal condensation in the presumptive primordium for tympanic ring in E13.5 *Wnt1-Cre/+;COUP-TFII$^{flox/-}$* mutant. Adjacent frontal sections through otic region from early E13.5 mouse heads were immunostained with antibodies against COUP-TFII, β-gal and Sox9. The spatial distribution of β-gal-positive cells was indistinguishable between *Wnt1-Cre/+;COUP-TFII$^{flox/+}$* controls (b') and *Wnt1-Cre/+;COUP-TFII$^{flox/flox}$* mutants (b''). Compared to controls, the Sox9 expression (white arrow) was also

dramatically up-regulated in the corresponding β-gal-positive region medial to the EAM before mesenchymal condensation in *Wnt1-Cre/+;COUP-TFII*$^{flox/-}$ mutants (c'' and d''). Corresponding Sox9-expressing area is indicated by yellow arrow in b-b''. Higher magnification of the Sox9-expressing area in c-c'' are shown in d-d'', respectively. Nuclei were stained with DAPI (blue in a-d'' and grey in e-e''). Scale bar = 100 μm.

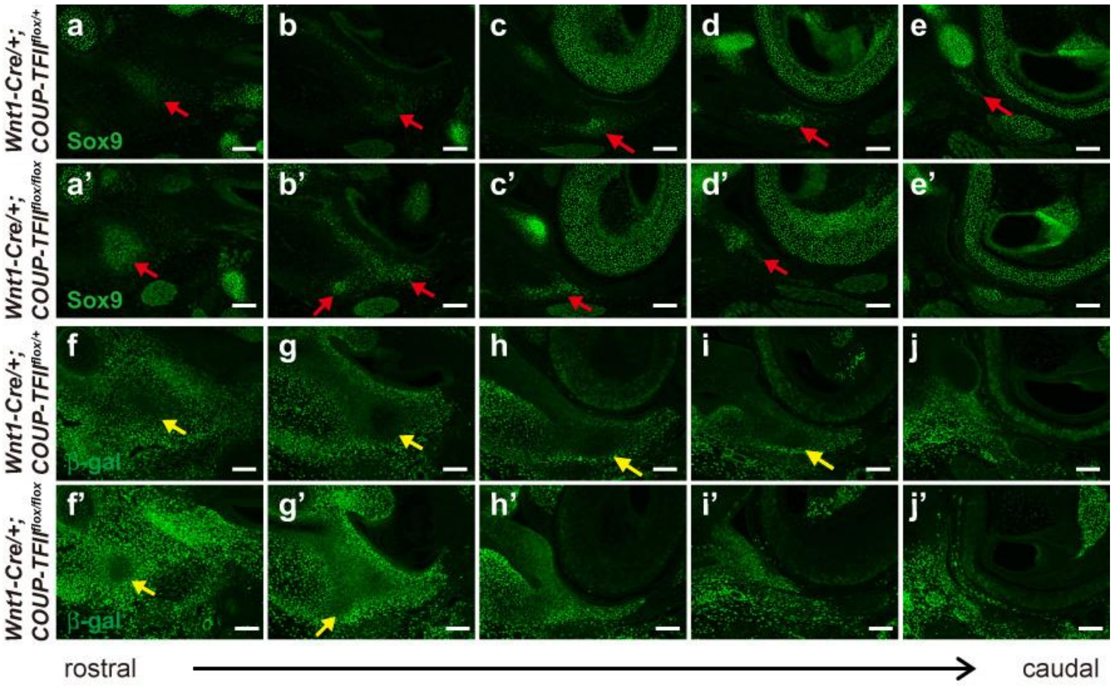

Fig. S2. Up-regulation of Sox9 in E14.5 *Wnt1-Cre/+;COUP-TFII*$^{flox/flox}$ mutants. Adjacent frontal sections through the otic region from E14.5 heads were immunostained with anti-Sox9 and anti-β-gal antibodies. (a-e') At E14.5, differential expression of Sox9 was detected in the mesenchyme of developing tympanic ring (red arrow). Sox9 was weakly expressed in the mesenchyme of rostral tympanic ring (a-b), whereas high level of Sox9 was detected in the mesenchyme of caudal tympanic ring (c-e) in *Wnt1-Cre/+;COUP-TFII*$^{flox/+}$ controls. Aberrant up-regulation of Sox9 in the

rostral part of developing tympanic ring was observed in *Wnt1-Cre/+;COUP-TFII*$^{flox/flox}$ mutants compared with the controls. Moreover, those Sox9-expressing mesenchymal cells further exhibited abnormal expansion with a bifurcated end in E14.5 *Wnt1-Cre/+;COUP-TFII*$^{flox/flox}$ mutants (a'-b'). (f-j') β-gal expression was broadly detected in cranial mesenchyme in both *Wnt1-Cre/+;COUP-TFII*$^{flox/+}$ controls (f-j) and *Wnt1-Cre/+;COUP-TFII*$^{flox/flox}$ mutants (f'-j'). However, the intensity of β-gal staining was decreased in the corresponding Sox9-expressing regions (yellow arrow). Scale bar = 100 μm.

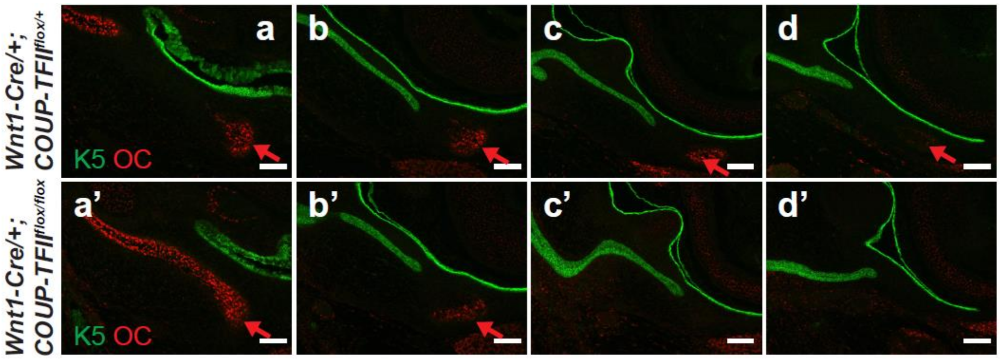

Fig. S3. The expression level of OC was not affected in E16.5 *Wnt1-Cre/+;COUP-TFII*$^{flox/flox}$ mutant. Serial frontal head sections through middle ear region from E16.5 controls (a-d) and *Wnt1-Cre/+;COUP-TFII*$^{flox/flox}$ mutants (a'-d') were immunostained with epithelial cell marker K5 (green) and osteoblast terminal differentiation marker OC (red) to designate the location of EAM and the bony part of tympanic ring (red arrow), respectively. Even though the mutant tympanic ring (a'-b') was shorter than control (a-d), the expression level of OC was comparable between the developing tympanic rings of control and *Wnt1-Cre/+;COUP-TFII*$^{flox/flox}$ embryos. Scale bar = 100 μm.

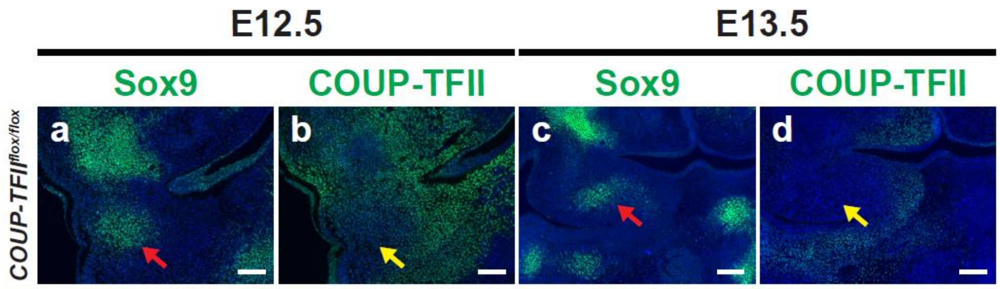

Fig. S4. Relative COUP-TFII protein levels in the melleal primordium and surrounding mesenchyme. Frontal sections through malleal primordium from E12.5 and E13.5 control mouse heads were immunostained with antibodies against Sox9 and COUP-TFII. While Sox9 (green, a) was detected in the developing malleal primordium (red arrow), the level of COUP-TFII (green, b) was lower in Sox9-expressing cells (yellow arrow) compared to those in the surrounding mesenchyme at E12.5. At 13.5, COUP-TFII was further down-regulated in both the developing malleus and the neighboring mesenchyme. Nuclei were stained with DAPI (blue). Scale bar = 100 µm.
